# Acute toxicity, brine shrimp cytotoxicity and relaxant activity of fruits of *callistemon citrinus *curtis

**DOI:** 10.1186/1472-6882-11-99

**Published:** 2011-10-24

**Authors:** Niaz Ali, Ghayour Ahmed, Syed Wadood Ali Shah, Ismail Shah, Mehreen Ghias, Imran Khan

**Affiliations:** 1 Department of Pharmacology, Institute of Basic Medical Sciences, Khyber Medical University, Peshawar, KPK, Pakistan; 2 Department of Pharmacy, University of Malakand, Chakdara, Dir, KPK, Pakistan; 3 Department of Biotechnology, University of Malakand, Chakdara, Dir, KPK, Pakistan

## Abstract

**Background:**

*Callistemon citrinus *Curtis belongs to family Myrtaceae that has a great medicinal importance. In our previous work, fruits of *Callistemon citrinus *were reported to have relaxant (antispasmodic) activity. The current work describes the screening of fractions of the crude methanol extract for tracing spasmolytic constituents so that it shall help us for isolation of bioactive compounds. Acute toxicity and brine shrimp cytotoxicity of crude methanol extract are also performed to standardize it.

**Methods:**

The crude methanol extract was obtained by maceration with distilled water (500 ml) three times and fractionated successively with *n-*hexane, chloroform, ethyl acetate and *n-*butanol (300 ml of each solvent). Phytochemical analysis for crude methanol extract was performed. Acute toxicity studies were performed in mice. Brine shrimp cytotoxicity studies were performed to determine its cytotoxicity and standardize it. In other series of experiments, rabbits' jejunum preparations were used in screening for possible relaxant activities of various fractions. They were applied in concentrations of 0.01, 0.03, 0.1, 0.3, 1.0, 3.0, 5.0 and 10.0 mg/ml on spontaneous rabbits' jejunum preparations. In similar fashion, fractions were also tested on KCl (80 mM) -induced contractions. Calcium chloride curves were constructed in K-rich Tyrode's solution. The effects of various fractions were tested on calcium chloride curves at concentrations 1.0, 3.0, 5.0 and 10.0 mg/ml. Curves of verapamil used as reference drug at concentration 0.1 μM and 0.3 μM were also constructed. The curves were compared with their respective controls for possible right shift.

**Results:**

Methanol extract tested strongly positive for saponins and tannins. However, it tested mild positive for presence of proteins, amino acids, carbohydrates and phenolic compounds. LD_50 _value for crude methanol extract is 476.25 ± 10.3 (470-481, n = 4) mg/ml. Similarly, EC_50 _value for brine shrimp cytotoxicity is 65.5 ± 7.28 (60.8- 69.4, n = 4) mg/ml. All the fractions relaxed the spontaneous and KCl-induced contractions. EC_50 _values (mg/ml) for effects of ethyl acetate fraction on spontaneous and KCl induced contractions are 2.62 ± 0.78 (2.15-3.0, n = 4) and 3.72 ± 0.86 (3.38-4.28, n = 4) respectively. Respective EC_50 _values (mg/ml) for *n-*butanol fraction are 3.59 ± 0.2(3.07-3.9, n = 4) for spontaneous, and 5.57 ± 0.2 (5.07-6.11, n = 4) for KCl- induced contractions. EC_50 _value for control calcium chloride curve (without extract) is -2.73 ± 0.19 (-2.6 - -2.81, n = 4) while EC_50 _for curves treated with 5.0 mg/ml of chloroform is -2.22 ± 0.02 (-2.16 - -2.3, n = 4). EC_50 _value for ethyl acetate treated (1.0 mg/ml) tissues is -1.95 ± 0.10 (-1.88 - -2.0, n = 4) *vs*. control EC_50 _= -2.71 ± 0.08 (-2.66 - -2.76, n = 4). All the fractions, except *n-*hexane, showed a right shift like that of verapamil (EC_50 _= -1.72 ± 0.15 (-1.62 - -1.8, n = 4) vs. Control EC_50 _= -2.41 ± 0.06 (-2.38 - - 2.44, n = 4), a standard drug that blocks voltage operated calcium channels.

**Conclusion:**

Relaxant constituents were more concentrated in ethylacetate fraction followed by chloroform, *n -*butanol and aqueous fractions that warrant for its isolation. The crude methanol extract is safe at concentration 250 mg/ml or below and results of brine shrimp cytotoxicity assay imply the plant specie may be a source of cytotoxic agents.

## Background

Family Myrtaceae consists of 3800 species, which are distributed in 140 genera. The plants are mostly found in tropical and subtropical regions of the world [1].The genus *Callistemon *belongs to family Myrtaceae. The plants of this genus are small evergreen beautiful trees and shrubs, which contain approximately 34 species. The cylindrical brush like flowers resemble traditional bottle brush, hence, the plant is commonly named as bottle brush [2].The leaves of *Callistemon *species are aromatic and lanceolate, mostly 40-70 mm long and 3-6 mm wide. The flowers are borne in spikes of about 40-150 mm long with prominent red stamens while its petals are tiny, pale or greenish colored [3]. Mostly, the species of this genus are used for ornamental purpose. However, species of the genus are sources of insecticidal, antibacterial and antifungal bioactive compounds as well [4]. Beside these activities, the genera have also some additional medicinal properties like antithrombotic, repellent, nematocidal, larvicidal and pupicidal activities [5]. Traditionally, in Chinese medicine, *Callistemon viminalis *is used for the treatment of hemorrhoids [6]. *Callistemon *species are also used for weed control, as bio indicators for environmental management, and a source of essential oil production as well. Phytochemically, the essential oils of *Callistemon citrinus *and *C. viminalis *contain α-pinene, β-pinene, α-terpinene, 1, 8-cineole, linalool, trans-pinocarveol, terpinen-4-ol, geraniol and α-terpineol that has showed antibacterial activity [3,7]. Preliminary screening of *Callistemon linearis *has revealed the presence of glycosides, saponins, flavonoids, phenolic compounds, phytosterols and carbohydrates. Hydro distillation of volatile oils mainly contain *n*-De-3-ene, 3-carene, 1, 8-cineole, γ-terpinene [2]. The Stem bark of *Callistemon rigidus *has led to the isolation of scirpusin B and piceatannol that have inhibitory activity on mouse alpha amylase [6]. In our first attempt for pharmacological screening of fruits of *Callistemon citrinus*, we have reported relaxant activity of its crude methanol extract on rabbits' jejunum preparations with possible mechanism through voltage operated calcium channels [8]. Objective of our current work is to screen the various fractions of crude methanol extract of *Callistemon citrinus*, and to know fraction(s) in which are concentrated the relaxant constituents. So that it shall make other scientists convenient for activity guided isolation of bioactive compounds. Acute toxicity and cytotoxic activities have also been performed to standardize the crude methanol extract of *C. citrinus *fruits.

## Methods

### Collection, extraction and fractionation of plant materials

Fresh fruits of *Callistemon citrinus *were collected in July 2009 from the campus of University of Malakand, KPK, Pakistan. The fruits were subjected to shade drying. The plant voucher specimen (CC-01-2009) was identified by Professor Dr. Jehandar Shah, vice chancellor Shaheed Benazir Bhutto University, Sheringal Dir Upper, Sheringal. The fruits were grinded to fine powder. The powdered materials (4.0 Kg) were soaked in commercial grade methanol (6 Liters) for 4 days. The materials were filtered through ordinary filter paper. The process was repeated thrice. The filtrates were combined and concentrated, at 40°C under reduced pressure, using a rotary evaporator till a brownish crude extract (400.0 g) was obtained. The brownish extract (350.0 g) was dissolved in 500 ml distilled water and was successively fractionated with (300 ml of each solvent, three times each) *n*-hexane, chloroform, ethyl acetate and *n*-butanol. Each fraction was treated as described above yielding corresponding fractions upon evaporation as *n*-hexane (30.0 g), chloroform (70.0 g), ethyl acetate (120.0 g), *n*-butanol (60.0 g) and residual aqueous fraction (70.0 g).

### Drugs and animals

All solutions were prepared on same day of experiments. Analytical grade chemicals were used in the experiments. Acetylcholine was purchased from BDH chemicals, Poole, England. Rests of the chemicals were purchased from E Merck Germany. Rabbits of either sex (weighing 1.4-2.0 Kg) were used in experiments (n = 4 each) as per "Byelaws of the University of Malakand 2008, Scientific Procedures, Issue I". The animals had a free access to water. However, they were starved 24 hours prior to start of experiments. Ethical Committee of Department of Pharmacy, University of Malakand approved the study protocols.

#### Data recordings

Intestinal responses were recorded with help of a Force Transducer (Model No: MLT 0210/A Pan Lab S.I.), connected with Power lab (Model No: 4/25 T) AD Instruments, Australia. Bridge Pod Amplifier was used for amplification of responses. Setting parameters were at range of 20 mv, Low pass 5Hz × 10 gain using input 1, rate 40/S.

#### Statistical analysis and interpretation of data

Statistical analysis was performed at 95% confidence interval. P value equal to or less than 0.05 was considered as significant. Chart 5 supplied with the power Lab was used to interpret the data. Microsoft XL sheet was used to calculate mean values. Graph Pad prism was used to calculate mean, SEM and draw the curves for LD_50_, EC_50 _shift.

#### Preliminary phytochemical screenings

Preliminary phytochemical screenings were performed for presence of saponins, tannins, carbohydrates, steroids, proteins, amino acids, phenolic compounds and anthraquinone glycosides [9,10].

#### Acute toxicity studies

Acute toxicity study on crude methanol extract was performed using mice model. Lorke (1983) method was used for acute toxicity study. The crude methanol extract was administered intraperitoneally (i.p) in mice [11]. Mice of either sex were fasted overnight. In the first stage of the preliminary screening, 3 groups, each of 3 mice were treated with the crude methanol extract at doses of 10, 100 and 1000 mg/kg (i.p) for the determination of the lethal range. In the second stage, another 4 groups of 3 mice each were further treated with the extract at doses of 125, 250, 400 and 600 mg/kg. Animals were continuously observed for 24 hours, after dosing, for study of acute toxicity. In each group, numbers of deaths were recorded within 24 hours. LD_50 _values were calculated.

#### Brine shrimp cytotoxicity study

Brine shrimp lethality bioassay was performed to investigate the cytotoxicity of crude methanol extract of *Callistemon citrinus*. Brine shrimp (*Artemia salina*) eggs were hatched using a conical shaped vessel (capacity = 1L) containing sterile artificial seawater (sea salt 38.0 g/L; adjusted pH 8.5) [12]. They were kept for 48 hours with continuous aeration. After hatching, active nauplii free from egg shells, were collected from brighter portion of the hatching chamber. Dimethyl sulfoxide (DMSO, 1.0 ml) was used for the preparation of different concentrations (1000, 100, 10 μg/ml) of sample extract, in triplicates. After evaporation of vehicle solvent, each test tube was introduced with ten brine shrimp larvae (10 nauplii). All test tubes were maintained at room temperature for 24 hours. The numbers of surviving and dead shrimps were counted. Percentage mortality was determined. Each concentration was triplicated. EC_50 _were calculated using graph pad prism software.

#### Effects on spontaneous rabbits' jejunum preparations and KCl- induced contractions

Rabbits were given a blow on cervix for cervical dislocation. Their abdomens were opened. Pieces of jejunum were removed and placed in Tyrode's solution aerated with carbogen gas (95% O_2_:5% CO_2_). Constituents and concentrations (mM) used in Tyrode's solution were KCl 2.68, NaCl 136.9, MgCl_2 _1.05, NaHCO_3 _11.90, NaH_2 _PO_4 _0.42, CaCl_2 _1.8 and glucose 5.55. The mesentery was removed. Pieces of about 1-1.5 cm length were mounted in 10 ml tissue bath at control temperature 37 ± 1°C. After stabilization, various fractions (*n*-hexane, chloroform, ethyl acetate, *n*-butanol and aqueous) of crude methanol extract were tried at concentrations 0.01, 0.03, 0.1, 0.3, 1.0, 3.0, 5.0 and 10.0 mg/ml [13-16]. Effects were recorded. Sustained contractions were produced in rabbits' jejunum preparations by KCl (80 mM). The fractions were tried in similar concentrations. Its effects were noted.

#### Effects on calcium chloride curves in decalcified tissues

In order to explain the possible mode of action, the tissues were successively decalcified in K-Normal Tyrode's solution and K Rich Tyrode's solution [13-16]. Earlier, the tissues were stabilized in normal Tyrode's solution for about 20 minutes. The composition of normal Tyrode's solution (mM) was KCl 2.68, NaCl 136.9, MgCl_2 _1.05, NaHCO_3 _11.90, NaH_2 _PO_4 _0.42, CaCl_2 _1.8 and glucose 5.55. Constituents and concentration (mM) of K Rich Tyrode's solution was KCl 50, NaCl 91.04, MgCl_2 _1.05, NaHCO_3 _11.90, NaH_2 _PO_4 _0.42, glucose 5.55 and EDTA 0.1. Control (in absence of test fractions) calcium chloride curves were constructed in decalcified tissues. The tissues were then exposed to known concentrations of fractions. An incubation period of 1 hour was given to same tissues so that test extract could produce possible calcium channel blocking effects. Calcium chloride curves were reconstructed to observe the effects of test extract. EC_50_, in the presence of extract, were calculated and compared with EC_50 _of control curves (absence of extract). In other series of experiments, in similar manners, curves were constructed for calcium chloride in presence and absence of known concentrations of verapamil. EC_50 _were calculated and compared with control curves for possible right shift. Curves of the fractions were compared with curves of verapamil, a standard calcium channel blocker for voltage operated calcium channels.

## Results and discussion

**Figure 1:**
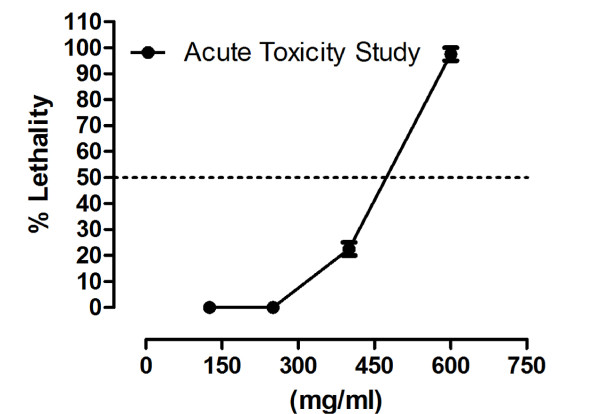
**Results of crude methanol extract of fruits of *Callistemon citrinus *for acute toxicity study in mice**.

**Figure 2:**
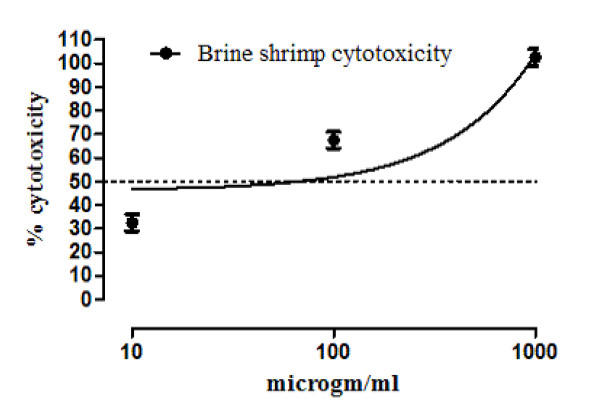
**Brine shrimp cytotoxicity of crude methanol extract of *Callistemon citrinus***.

**Figure 3:**
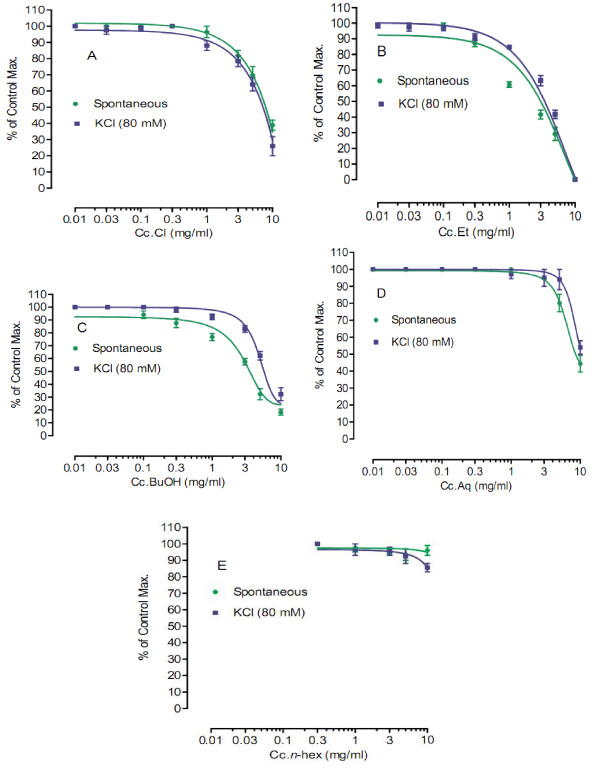
**(A): Effects of Chloroform fraction of *Callistemon citrinus *(Cc.Cl) on spontaneous and KCl-induced contractions**. (B): Effects of ethyl acetate fraction of *Callistemon citrinus *(Cc.Et) on spontaneous and KCl-induced contractions. (C): Effects of *n-*butanol fraction of *Callistemon citrinus *(Cc.BUOH) on spontaneous and KCl-induced contractions. (D): Effects of aqueous fraction of *Callistemon citrinus *(Cc.Aq) on spontaneous and KCl-induced contractions. (E): Effects of *n-*hexane fraction of *Callistemon citrinus *(Cc.*n*-hex) on spontaneous and KCl-induced contractions.

**Figure 4:**
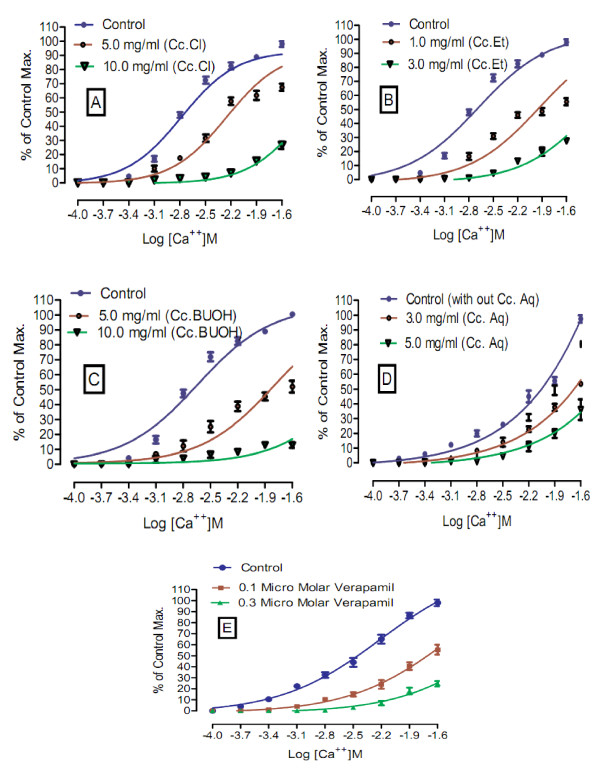
**(A): Effects of chloroform fraction of *Callistemon citrinus *(Cc.Cl) on calcium chloride curves versus control**. (B): Effects of ethylacetate fraction of *Callistemon citrinus *(Cc.Et) on calcium chloride curves versus control. (C): Effects of *n-*butanol fraction of *Callistemon citrinus *(Cc.BUOH) on calcium chloride curves versus control. (D): Effects of aqueous fraction of *Callistemon citrinus *(Cc.Aq) on calcium chloride curves versus control. (E): Effects of O.1 μM Verapamil on calcium chloride curves versus control.

Upon preliminary phytochemical screenings, methanol extract tested strongly positive for saponins and tannins. However, it tested positive for presence of proteins, amino acids, carbohydrates and phenolic compounds (Table 1). Further, it tested negative for presence of steroids, alkaloids, flavonoids and anthraquinone glycosides. In our earlier published report, the crude extract has tested positive for presence of terpenes (terpenoids) and saponins glycosides [8-10]. Results of acute toxicity studies are summarized in Table 2. In the initial stage of screening, all animals of group 3 died showing that the methanol extract is 100% lethal at the said concentration 1000 mg/kg. At dose 10 mg/kg (group 1) and 100 mg/kg (group 2), all animals survived. In the 2^nd ^phase of screening, animals of group 1 and group 2 survived, respectively at concentration 125 mg/kg and 250 mg/kg of crude methanol extract, while one mouse died at concentration of 400 mg. All animals in group 4 died. Their % lethality is expressed in Figure 1. LD_50 _value for the graph in Figure 1 is 476.25 ± 10.3 (470-481, n = 4) mg/ml. Similarly, results of brine shrimp cytotoxicity studies are expressed in Figure 2. EC_50 _value is 65.5 ± 7.28 (60.8 - 69.4, n = 4) mg/ml. It is, thus stated that the plant specie has good cytotoxic effect and, therefore, may be a source of anticancer constituents as there is positive correlation between the brine shrimp toxicity and human nasopharyngeal carcinoma [12]. Thus the brine shrimp cytotoxicity implies for presence of cytotoxic constituents as well. As per our previous reported work [8] that crude methanol extract possess relaxant activity on rabbits' jejunum preparations, the current work describes the screening of fractions of *Callistemon citrinus *for possible relaxant effects that may be helpful for other scientists for further work. According to Figure 3(A), which describes the results for effects of chloroform fraction, the EC_50 _value on spontaneous rabbits' jejunum preparations is 7.84 ± 0.16 (7.42-8.18, n = 4) mg/ml while EC_50 _for KCl -induced contractions at 7.0 ± 0.73 (6.63-7.42, n = 4) mg/ml. EC_50 _values (mg/ml) for effects of ethyl acetate fraction on spontaneous and KCl induced contractions are 2.62 ± 0.78 (2.15-3.0, n = 4) and 3.72 ± 0.86 (3.38-4.28, n = 4) respectively (Figure 3(B)). Respective EC_50 _values for *n-*butanol fraction are 3.59 ± 0.2 (3.07-3.9, n = 4) and 5.57 ± 0.2 (5.07-6.11, n = 4) mg/ml (Figure 3(C)). Similarly, respective EC_50 _values for aqueous fraction are 7.99 ± 0.3 (7.07-8.73, n = 4) and 9.62 ± 0.1 (9.47-9.79, n = 4) mg/ml (Figure 3(D)). EC_50 _values for *n-*hexane fraction were not calculated as its effects on spontaneous and KCl-induced contractions were very low. This fraction could not relax more than 15% of control response (Figure 3(E)). The contractile effects of the jejunum are due to free calcium levels of the tissues. The intracellular and extracellular calcium stores also exchange with each other that lead to periodic depolarization and repolarization of jejunum. The periodic depolarization and repolarization are responsible for the intestinal responses [13-15,17-21]. It is not necessary that relaxing effects on KCl induced contractions are always through calcium channels [13,14]; hence, the calcium chloride curves in decalcified tissues were constructed (Figure 4) [13-17]. Figure 4 (A) shows the effects of chloroform fraction on calcium chloride curves, EC_50 _[log (Ca^++^)M] value for control curve (without extract) is -2.73 ± 0.19 (-2.6 - -2.81, n = 4) while EC_50 _for curves treated with 5.0 mg/ml of chloroform is -2.22 ± 0.02 (-2.16 - -2.3, n = 4). EC_50 _value for ethylacetate treated (1.0 mg/ml) tissues is -1.95 ± 0.10 (-1.88 - -2.0, n = 4) *vs*. control EC_50 _= -2.71 ± 0.08 (-2.66 - -2.76, n = 4) (Figure 4 (B)). Similarly, for *n-*butanol fraction, EC_50 _values for control and 5 mg/ml of the fraction are, respectively, -2.71 ± 0.10 (-2.65 - -2.77, n = 4) and -1.82 ± 0.07 (-1.79 - -1.87, n = 4) (Figure 4 (C)). In case of aqueous fraction, EC_50 _for control = -2.09 ± 0.10 (-2.04 - -2.15, n = 4) (Figure 4 (D)), while EC_50 _value for aqueous fraction (3 mg/ml) = -1.66 ± 0.10 (-1.6 - -1.72, n = 4) (Figure 4 (D)). EC_50 _value for verapamil (0.1 μM) treated tissues = -1.72 ± 0.15 (-1.62 - -1.8, n = 4) vs. Control EC_50 _= -2.41 ± 0.06 (-2.38 - - 2.44, n = 4) (Figure 4 (E)). Thus all the fractions, except *n-*hexane, showed a right shift like that of verapamil, a standard drug that blocks voltage operated calcium channels. This suggests the presence of calcium antagonist compounds in the fractions of the crude methanol extract. It is noteworthy (according to our previous published data) that the crude methanol extract of the plant had shifted the calcium chloride curves to right at "concentration of 0.03 mg/ml of extract treated tissues (EC_50 _= -2.05 ± 0.05 (Log [Ca^++^] M *vs*. control EC_50 _= -2.5 ± 0.05 (Log [Ca^++^] M)" [8]. The current study on fractions of the crude methanol extract revealed that the calcium antagonist compounds were concentrated more in ethylacetate fraction that produced right shifted at 1 mg/ml, followed by aqueous fraction where it produced right shift at concentration 3.0 mg/ml. *n-*butanol and chloroform fractions showed a right shift at 5.0 mg/ml. It is thus concluded that the presence of calcium antagonists were more concentrated in ethylacetate fraction that opens a new window for phytochemists for isolation of bioactive calcium antagonists directly from the ethyl acetate fraction. The relaxant effect of fractions of *Callistemon citrinus *on rabbit's jejunum preparations may be attributed to the phytochemical constituents such as saponins, tannins and triterpenes present in the plant as also reported in previous studies [15,22-24]. The cytotoxic activity may be attributed to saponins as well [24].

**Table 1:** Phytochemical screening of crude methanol extract of *Callistemon citrinus*.

*S. No*	*Class of Phytochemicals*	*Results*
1	*Saponins*	*+++*
2	*Tannins*	*+++*
3	*Carbohydrates*	*+*
4	*Steroids*	*_*
5	*Proteins*	*+*
6	*Amino Acids*	*+*
7	*Phenolic Compounds*	*+ *
8	*Flavonoids*	*_*
9	*Anthraquinone glycosides*	*_*
10	*Alkaloids*	*_*

**Table 2:** Results of acute toxicity studies of crude methanol extract of *Callistemon citrinus *in mice.

1^st ^stage		Dose (mg/kg Body weight)		
	**Group1** (10 mg)	**Group 2** (100 mg)	**Group 3** (1000 mg)	
	All alive	All alive	All dead	
**2^nd ^stage**	**Group 1** (125 mg)	**Group 2** (250 mg)	**Group3** (400 mg)	**Group 4** (600 mg)
	All alive	All alive	1 died	All dead

## Conclusions

Relaxant constituents were more concentrated in ethylacetate fraction followed by chloroform, *n-*butanol and aqueous fractions that warrant for its isolation. The crude methanol extract is safe at concentrations 250 mg/ml or below and results of brine shrimp cytotoxicity assay imply that the plant specie may be a source of cytotoxic agents.

## Competing interests

The authors declare that they have no competing interests.

## Authors' contributions

NA: Data interpretation, preparation of the manuscript, answering the queries of the reviewers and formatting the final manuscript. GA: Data acquisition. SWAS: Data acquisition and helped in writing introduction section of the manuscript. IS:Data acquisition. MG: Data acquisition. IK: Data acquisition. All the authors have read and approved the final proof of the manuscript.

## Pre-publication history

The pre-publication history for this paper can be accessed here:

http://www.biomedcentral.com/1472-6882/11/99/prepub
